# Supplementary material for: Infectious stimuli promote malignant B-cell acute lymphoblastic leukemia in the absence of AID
Source: Nat Commun. 2019 Dec 5;10:5563. doi: 10.1038/s41467-019-13570-y (PMC6895129; doi:10.1038/s41467-019-13570-y)
Supplement: Supplementary file 1 — Supplementary Information [file 41467_2019_13570_MOESM1_ESM.pdf]

Supplementary Information

Infectious stimuli promote malignant B-cell acute lymphoblastic leukemia in the absence of AID

Rodríguez-Hernández, Opitz and Delgado et al.

Supplementary Figures

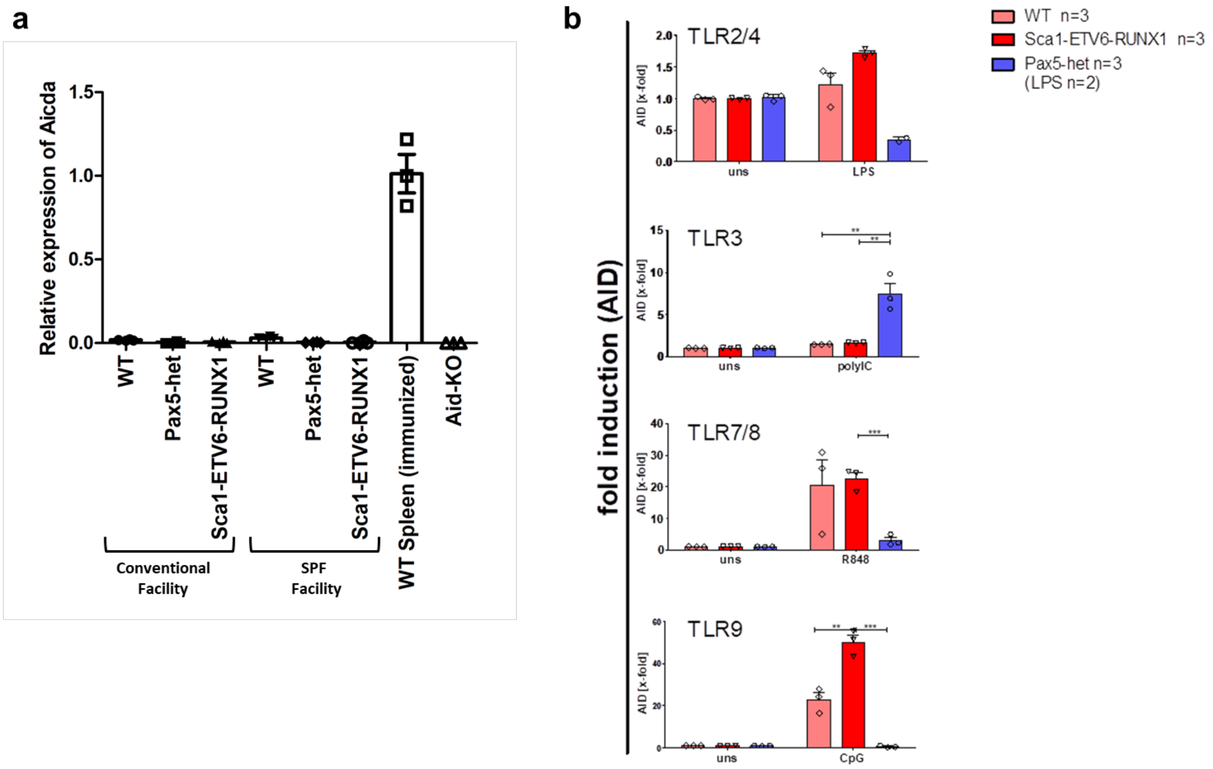

Supplementary Figure 1: Aid expression in preleukemic precursor B cells. a) Aid expression during natural infection driven mouse B-ALL. Relative expression of *Aid in BM preleukemic* precursor pro-pre-B cells sorted from control wild-type (WT), *Pax5-het*, *Sca1-ETV6-RUNX1* either housed under SPF conditions or exposed to natural infections (conventional facility). WT total spleen of an immunized mouse was used as a positive control. Precursor pro-pre-B cells sorted from *Aid-KO* mice were used as a negative control. Error bars represent the mean ± SD of three replicates. **b) Aid expression in murine preleukemic *Pax5-het*, and *Sca1-ETV6-RUNX1* pro-B cells after *in vitro* stimulation with TLR ligands.** Fold induction of Aid expression on RNA levels of murine pro-B cells of WT, *Sca1-ETV6-RUNX1* and *Pax5-het* mice after stimulation with different TLR ligands (TLR2/4 - LPS, TLR3 - polyIC, TLR7/8 - R848 and TLR9 - CpG) *in vitro*. Experiments were performed in 3 replicates, condition Pax5-het + LPS was performed in 2 replicates. Error bars represent the mean ± SD of three replicates. The expression levels were normalized to their unstimulated control. **p<0.01, ***p<0.001; Student´s two tailed t-test.

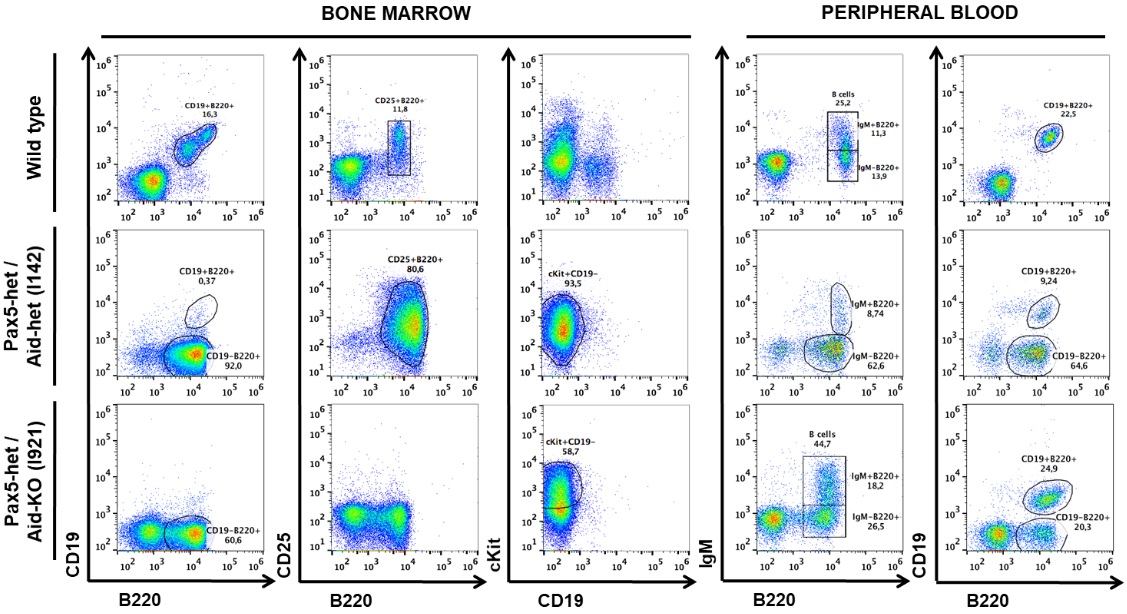

Supplementary Figure 2: Flow cytometric analysis of diseased *Pax5-het/Aid-het* mice and *Pax5-het/Aid-KO* mice. Representative plots of cell subsets are shown and compared to wild-type mice.

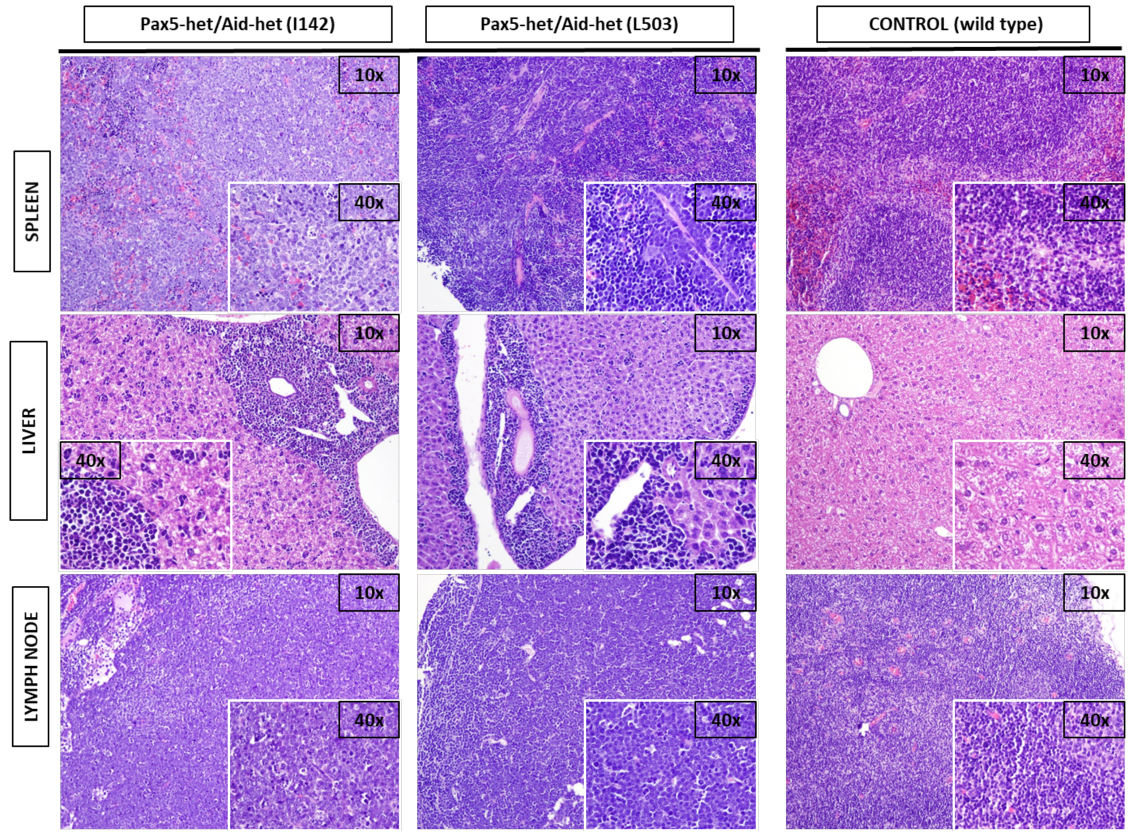

Supplementary Figure 3: Haematoxylin and eosin staining of tumour-bearing *Pax5-het/Aid-het* mice. H-E staining of WT mice and tumour-bearing *Pax5-het/Aid-het* mice showing infiltrating blast cells in spleen, liver, and lymph nodes. Loss of normal architecture resulting from effacement with cells morphologically resembling lymphoblast can be shown. Images are representative of 3 replicates. Scale bar represents 500 μm (=100X) for large panels and 100 μm (=400X) for inset.

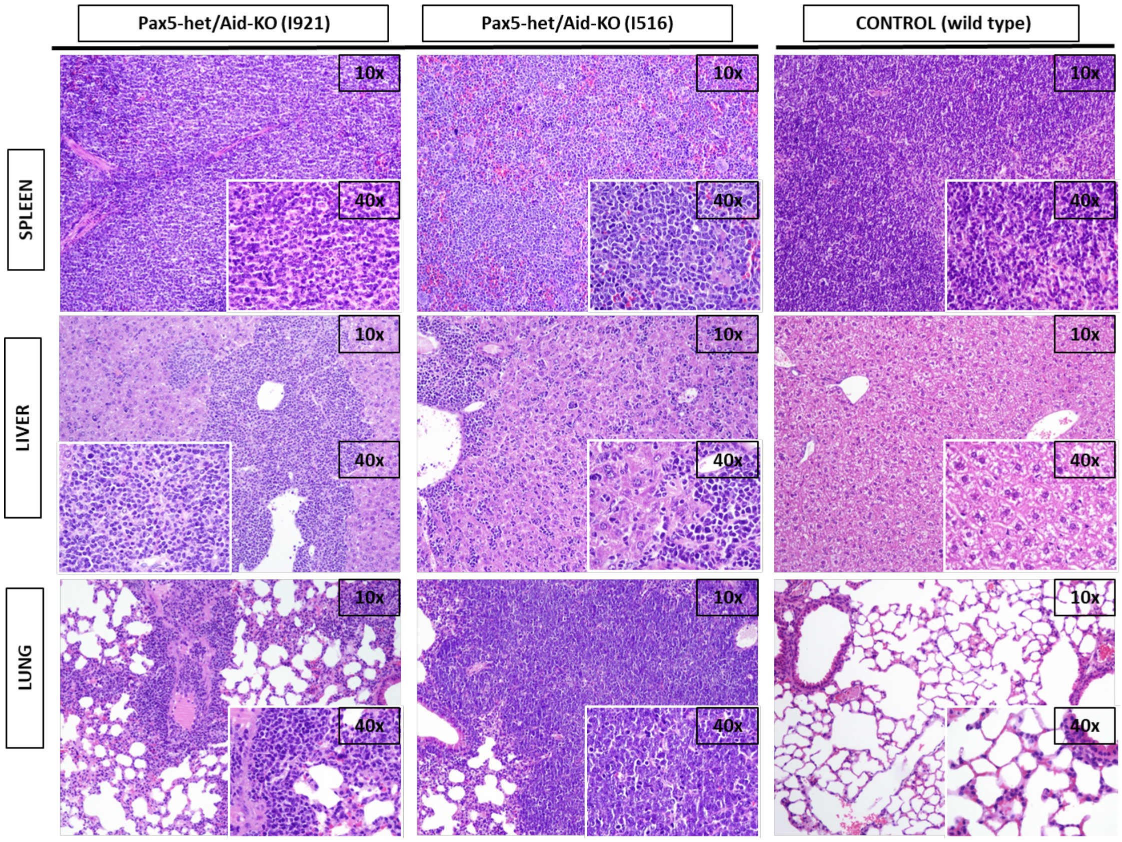

Supplementary Figure 4: Haematoxylin and eosin staining of tumour-bearing *Pax5-het/Aid-KO* mice. H-E staining of WT mice and tumour-bearing *Pax5-het/Aid-KO* mice showing infiltrating blast cells in spleen, liver, and lung. Loss of normal architecture resulting from effacement with cells morphologically resembling lymphoblast can be shown. Images are representative of 3 replicates. Scale bar represents 500 μm (=100X) for large panels and 100 μm (=400X) for inset.

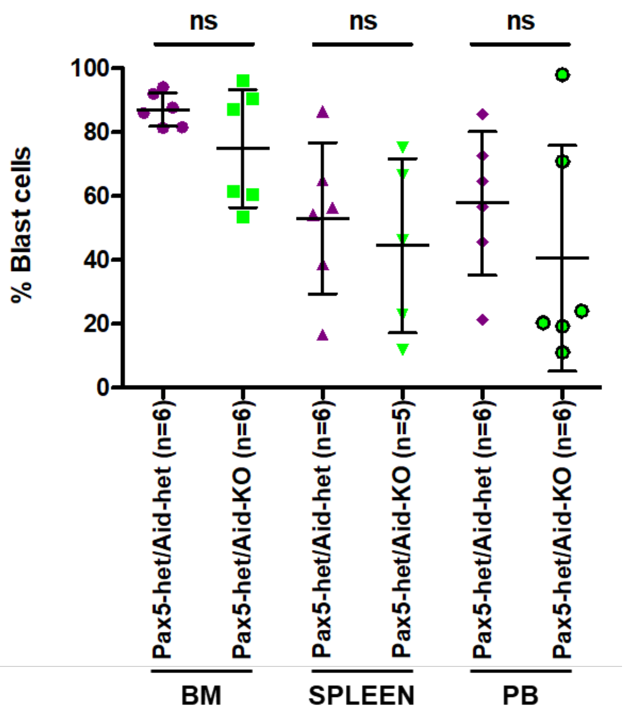

Supplementary Figure 5: Comparison of blast cell percentages in *Pax5-het/Aid-het* and *Pax5-het/Aid-KO mice*. The results showed that there are not statistical differences between both groups (Mann Whitney test). Error bars represent the mean ± SD of three replicates.

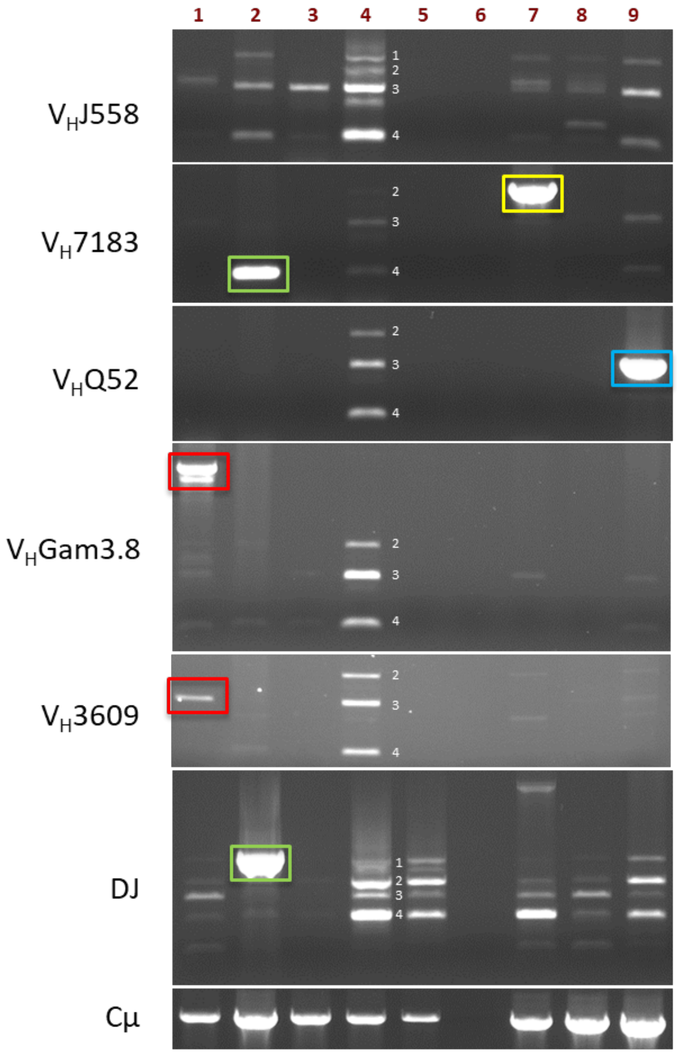

1. I921 (Pax5-het /Aid-KO)
2. I516 (Pax5-het /(Aid-KO)
3. V414 (Pax5-het/ Aid-KO)
4. B cells (Wild-type)
5. T cells (Wild-type)
6. H2O
7. L503 (Pax5-het /Aid-het)
8. I142 (Pax5-het/ Aid-het)
9. L517 (Pax5-het/ Aid-het)

Supplementary Figure 6: Immunoglobulin clonality in *Pax5-het/Aid-het* and *Pax5-het/Aid-KO* B-ALL. PCR analysis of immunoglobulin heavy-chain gene rearrangements in infiltrated BM of diseased *Pax5-het/Aid-het* mice and *Pax5-het/Aid-KO* mice. Thymocytes (T cells) were included as a negative control, and sorted CD19^{+} B-cells (B cells) from the spleens of healthy mice were included as a control for polyclonal rearrangements (indicated by numbers, 1-4) within the mature B-cell population. It can be seen that infiltrated tissues shown an increased clonality within their immunoglobulin repertoire (coloured squares). Source data are provided as a Source Data file.

a)

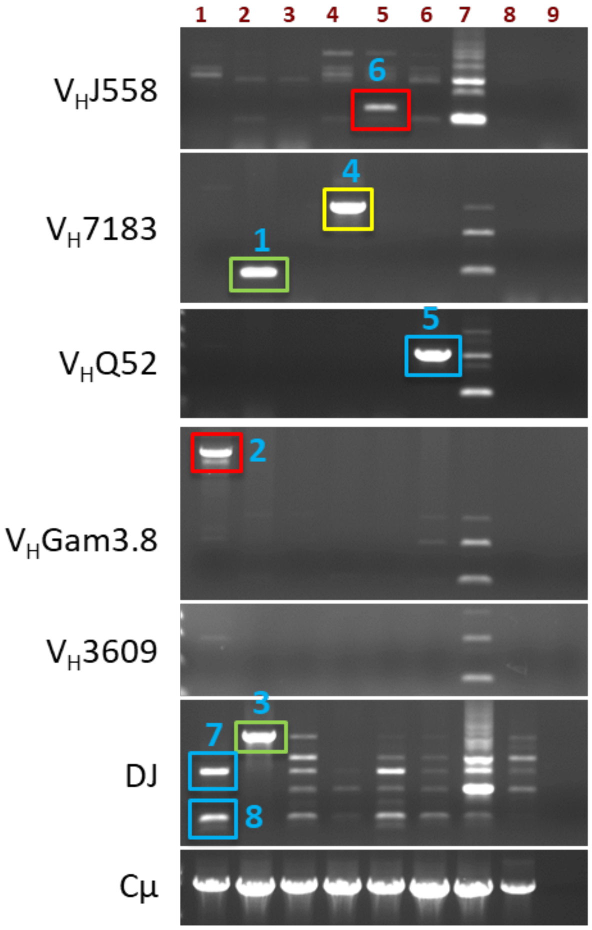

Bone Marrow samples:

1. I921 (Aid-/- ; Pax5+/-)
2. I516 (Aid-/- ; Pax5+/-)
3. V414 (Aid-/- ; Pax5+/-)
4. L503 (Aid+/- ; Pax5+/-)
5. I142 (Aid+/- ; Pax5+/-)
6. L517 (Aid+/- ; Pax5+/-)
7. B cells (Wild-type)
8. T cells (Wild-type)
9. H2O

b)

Band 1; 2-I516 (Aid-/- ; Pax5+/-)

Query= CVD001
Length=359

Sequences producing significant alignments:	Score (Bits)	E Value
IGHV5-2*01germline gene	31.4	0.017
IGHV5-2*02germline gene	31.4	0.017
IGHV5-6-2*01germline gene	31.4	0.017
IGHD3-3*01germline gene	10.3	98
IGHJ3*01germline gene	93.0	3e-23
IGHJ3*02germline gene	81.4	8e-20
IGHJ2*01germline gene	27.6	0.001

Domain classification requested: imgt
V-(D)-J rearrangement summary for query sequence (multiple equivalent top matches, if present, are separated by a comma):

Top V gene match	Top D gene match	Top J gene match	Chain type	stop codon	V-J frame	Productive	Strand
IGHV5-2*01,IGHV5-2*02,IGHV5-6-2*01	IGHD3-3*01	IGHJ3*01	VH	No	Out-of-frame	No	+

V-(D)-J junction details based on top germline gene matches:

V region end	V-D junction*	D region	D-J junction*	J region start
TGCAA	TTT	TAGGG	GG	CCTGG

*: Overlapping nucleotides may exist at V-D-J junction (i.e, nucleotides that could be assigned to either rearranging gene). Such nucleotides are indicated inside a parenthesis (i.e., (TACAT)) but are not included under the V, D or J gene itself.
Sub-region sequence details:

	Nucleotide sequence	Translation	Start	End
CDR3	GCAATTTTAGGGGGCCTGGTTTGCTTAC	AILGGLVCL	38	65

Alignment summary between query and top germline V gene hit:

	from	to	length	matches	mismatches	gaps	identity(%)
FR3-IMGT	13	37	25	20	5	0	80
CDR3-IMGT (germline)	38	41	4	4	0	0	100
Total			29	24	5	0	82.8

Alignments

```
                                     <-------FR3-IMGT--------><---------CDR3-IMGT-------->
                                      E  X  X  A  L  X  Y  C  A  I  L  G  G  L  V  C  L  L  G  P  R  D  S  G  H  C  L  C
                    Query_1      13  TGAGGNNNCNGCCTTGTNTTACTGTGCAATTTTAGGGGGCCTGGTTTGCTTACTGGGGCCAAGGGACTCTGGTCACTGTCTCTGCAG  99
V  82.8% (24/29)    IGHV5-2*01   264 TGAGGACACAGCCTTGTATTACTGTGCAA----------------------------------------------------------  292
                                      E  D  T  A  L  Y  Y  C  A
V  82.8% (24/29)    IGHV5-2*02   264 TGAGGACACAGCCTTGTATTACTGTGCAA----------------------------------------------------------  292
V  82.8% (24/29)    IGHV5-6-2*01 264 TGAGGACACAGCCTTGTATTACTGTGCAA----------------------------------------------------------  292
D  100.0% (5/5)     IGHD3-3*01   9   --------------------------------TAGGG--------------------------------------------------  13
J  100.0% (48/48)   IGHJ3*01     1   ---------------------------------------CCTGGTTTGCTTACTGGGGCCAAGGGACTCTGGTCACTGTCTCTGCAG  48
J  95.8% (46/48)    IGHJ3*02     1   ---------------------------------------CCTGGTTTGGTTAGTGGGGCCAAGGGACTCTGGTCACTGTCTCTGCAG  48
J  100.0% (14/14)   IGHJ2*01     12  --------------------------------------------------TACTGGGGCCAAGG-----------------------  25
```

```
Lambda     K      H
   1.10    0.333  0.549

Gapped
Lambda     K      H
   1.08    0.280  0.540

Effective search space used: 48598413
```

```
Total queries = 1
Total identifiable CDR3 = 1
Total unique clonotypes = 1
```

c)

Band 4; 4-L503 (Aid+/-; Pax5+/-)

Query= CVD007
Length=600

```
Score        E
Sequences producing significant alignments:                          (Bits)  Value

IGHV5-12-2*01germline gene                                            48.6    2e-07
IGHV5-12-2*02germline gene                                            48.6    2e-07
IGHV5-15*05germline gene                                              48.6    2e-07
IGHD1-1*02germline gene                                               14.1    10
IGHD1-3*01germline gene                                               14.1    10
IGHD6-1*01germline gene                                               14.1    10
IGHJ1*01germline gene                                                 100     2e-25
IGHJ1*02germline gene                                                 94.9    1e-23
IGHJ1*03germline gene                                                 94.9    1e-23
```

Domain classification requested: imgt

V-(D)-J rearrangement summary for query sequence (multiple equivalent top matches, if present, are separated by a comma):

Top V gene match	Top D gene match	Top J gene match	Chain type	stop codon	V-J frame	Productive	Strand
IGHV5-12-2*01,IGHV5-12-2*02,IGHV5-15*05	IGHD1-1*02,IGHD1-3*01,IGHD6-1*01	IGHJ1*01	VH	No	In-frame	Yes	+

V-(D)-J junction details based on top germline gene matches:

V region end	V-D junction*	D region	D-J junction*	J region start
AGACA	TAGGGGGGTCGA	TGGTAGC	CTCCTT	TACTG

*: Overlapping nucleotides may exist at V-D-J junction (i.e, nucleotides that could be assigned
to either rearranging gene). Such nucleotides are indicated inside a parenthesis (i.e., (TACAT))
but are not included under the V, D or J gene itself.

Sub-region sequence details:

	Nucleotide sequence	Translation	Start	End
CDR3	GCAAGACATAGGGGGGTCGATGGTAGCCTCCTTTACTGGTACTTCGATGTC	ARHRGVDGSLLYWYFDV	37	87

Alignment summary between query and top germline V gene hit:

	from	to	length	matches	mismatches	gaps	identity(%)
FR3-IMGT	9	36	28	25	3	0	89.3
CDR3-IMGT (germline)	37	44	8	8	0	0	100
Total			36	33	3	0	91.7

Alignments

```
<---------FR3-IMGT--------><-------------------CDR3-IMGT------------------>
          S  X  X  X  A  M  Y  Y  C  A  R  H  R  G  V  D  G  S  L  L  Y  W  Y  F  D  V  W  G  A  G
```

```
                          Query_1       9    GTCTGANGANNCGGCCATGTATTACTGTGCAAGACATAGGGGGGTCGATGGTAGCCTCCTTTACTGGTACTTCGATGTCTGGGGCGCAGG  98
V  91.7% (33/36)          IGHV5-12-2*01 261  GTCTGAGGACACGGCCATGTATTACTGTGCAAGACA------------------------------------------------------  296
                                               S  E  D  T  A  M  Y  Y  C  A  R
V  91.7% (33/36)          IGHV5-12-2*02 228  GTCTGAGGACACGGCCATGTATTACTGTGCAAGACA------------------------------------------------------  263
V  91.7% (33/36)          IGHV5-15*05   261  GTCTGAGGACACGGCCATGTATTACTGTGCAAGACA------------------------------------------------------  296
D  100.0% (7/7)           IGHD1-1*02    14   ------------------------------------------------TGGTAGC-----------------------------------  20
D  100.0% (7/7)           IGHD1-3*01    14   ------------------------------------------------TGGTAGC-----------------------------------  20
D  100.0% (7/7)           IGHD6-1*01    8    ---------------------------------------------------TAGCCTC--------------------------------  14
J  100.0% (52/52)         IGHJ1*01      2    -------------------------------------------------------------TACTGGTACTTCGATGTCTGGGGCGCAGG  30
J  98.1% (51/52)          IGHJ1*02      2    -------------------------------------------------------------TACTGGTACTTCGATGTCTGGGGCGCAGG  30
J  98.1% (51/52)          IGHJ1*03      2    -------------------------------------------------------------TACTGGTACTTCGATGTCTGGGGCACAGG  30


                                               T  T  V  T  V  S  S
                          Query_1       99   GACCACGGTCACCGTCTCCTCAG  121
J  100.0% (52/52)         IGHJ1*01      31   GACCACGGTCACCGTCTCCTCAG  53
J  98.1% (51/52)          IGHJ1*02      31   GACCACGGTCACCGTTTCCTCAG  53
J  98.1% (51/52)          IGHJ1*03      31   GACCACGGTCACCGTCTCCTCAG  53
```

```
Lambda      K        H
    1.10    0.333    0.549

Gapped
Lambda      K        H
    1.08    0.280    0.540

Effective search space used: 83511605
```

```
Total queries = 1
Total identifiable CDR3 = 1
Total unique clonotypes = 1
```

d)

Band 5; 6-L517 (Aid+/- ; Pax5+/-)

Query= CVD009
Length=793

Sequences producing significant alignments:	Score (Bits)	E Value
IGHV2-4*01germline gene	76.6	1e-15
IGHV2-5*01germline gene	76.6	1e-15
IGHV2-5-1*01germline gene	76.6	1e-15
IGHD2-5*01germline gene	33.4	2e-05
IGHD2-6*01germline gene	33.4	2e-05
IGHD2-10*01germline gene	27.6	0.001
IGHJ2*01germline gene	89.1	9e-22
IGHJ2*02germline gene	83.4	5e-20
IGHJ2*03germline gene	83.4	5e-20

Domain classification requested: imgt

V-(D)-J rearrangement summary for query sequence (multiple equivalent top matches, if present, are separated by a comma):

Top V gene match	Top D gene match	Top J gene match	Chain type	stop codon	V-J frame	Productive	Strand
IGHV2-4*01,IGHV2-5*01,IGHV2-5-1*01	IGHD2-5*01,IGHD2-6*01	IGHJ2*01	VH	Yes	Out-of-frame	No	+

V-(D)-J junction details based on top germline gene matches:

V region end	V-D junction*	D region	D-J junction*	J region start
AAAAA	AAATTCCCCTC	CCTACTATAGTAACTAC	GTGG	TACTT

*: Overlapping nucleotides may exist at V-D-J junction (i.e, nucleotides that could be assigned
to either rearranging gene). Such nucleotides are indicated inside a parenthesis (i.e., (TACAT))
but are not included under the V, D or J gene itself.

Sub-region sequence details:

	Nucleotide sequence	Translation	Start	End
CDR3	GCCAAAAAAAATTCCCCTCCCTACTATAGTAACTACGTGGTACTTTGACTAC	AKKNSPPYYSNYVVL*L	60	111

Alignment summary between query and top germline V gene hit:

	from	to	length	matches	mismatches	gaps	identity(%)
FR3-IMGT	20	59	40	40	0	0	100
CDR3-IMGT (germline)	60	67	8	8	0	0	100
Total			48	48	0	0	100

Alignments

```
                                        <---------------FR3-IMGT---------------><---------------------CDR3-IMGT-------------------
                                         N  S  L  Q  A  D  D  T  A  I  Y  Y  C  A  K  K  N  S  P  P  Y  Y  S  N  Y  V  V  L  *  L
                        Query_1    20   GAACAGTCTGCAAGCTGATGACACTGCCATATACTACTGTGCCAAAAAAAATTCCCCTCCCTACTATAGTAACTACGTGGTACTTTGACT  109
V  100.0% (48/48)   IGHV2-4*01     246  GAACAGTCTGCAAGCTGATGACACTGCCATATACTACTGTGCCAAAAA------------------------------------------  293
                                         N  S  L  Q  A  D  D  T  A  I  Y  Y  C  A  K
V  100.0% (48/48)   IGHV2-5*01     246  GAACAGTCTGCAAGCTGATGACACTGCCATATACTACTGTGCCAAAAA------------------------------------------  293
V  100.0% (48/48)   IGHV2-5-1*01   246  GAACAGTCTGCAAGCTGATGACACTGCCATATACTACTGTGCCAAAAA------------------------------------------  293
D  100.0% (17/17)   IGHD2-5*01     1    -----------------------------------------------------------CCTACTATAGTAACTAC--------------  17
D  100.0% (17/17)   IGHD2-6*01     1    -----------------------------------------------------------CCTACTATAGTAACTAC--------------  17
D  94.1% (16/17)    IGHD2-10*01    1    -----------------------------------------------------------CCTACTATGGTAACTAC--------------  17
J  100.0% (46/46)   IGHJ2*01       3    --------------------------------------------------------------------------------TACTTTGACT  12
J  97.8% (45/46)    IGHJ2*02       3    --------------------------------------------------------------------------------TACTTTGACT  12
J  97.8% (45/46)    IGHJ2*03       1    --------------------------------------------------------------------------------TACTTTGACT  10

                                        ->
                                          L  G  P  R  H  H  S  H  S  L  L
                        Query_1    110  ACTGGGGCCAAGGCACCACTCTCACAGTCTCCTCAG  145
J  100.0% (46/46)   IGHJ2*01       13   ACTGGGGCCAAGGCACCACTCTCACAGTCTCCTCAG  48
J  97.8% (45/46)    IGHJ2*02       13   ACTGGGGCCAAGGCACCTCTCTCACAGTCTCCTCAG  48
J  97.8% (45/46)    IGHJ2*03       11   ACTGGGGCCAAGGCACCAGTCTCACAGTCTCCTCAG  46
```

```
Lambda      K        H
    1.10    0.333    0.549

Gapped
Lambda      K        H
    1.08    0.280    0.540

Effective search space used: 111738820
```

```
Total queries = 1
Total identifiable CDR3 = 1
Total unique clonotypes = 1
```

e)

Band 6; 5-I142 (Aid+/-; Pax5+/-)

Query= CVD012
Length=465

Sequences producing significant alignments:	Score (Bits)	E Value
IGHV1-54*02germline gene	43.9	4e-06
IGHV1-55*01germline gene	40.8	3e-05
IGHV1-62-1*01germline gene	40.8	3e-05
IGHD5-2*01germline gene	16.1	2.1
IGHD5-3*01germline gene	16.1	2.1
IGHD5-4*01germline gene	16.1	2.1
IGHJ3*01germline gene	93.0	4e-23
IGHJ3*02germline gene	81.4	1e-19
IGHJ2*01germline gene	27.6	0.002

Domain classification requested: imgt

Note that your query represents the minus strand of a V gene and has been converted to the plus strand. The sequence positions refer to the converted sequence.

V-(D)-J rearrangement summary for query sequence (multiple equivalent top matches, if present, are separated by a comma):

Top V gene match	Top D gene match	Top J gene match	Chain type	stop codon	V-J frame	Productive	Strand
IGHV1-54*02	IGHD5-2*01,IGHD5-3*01,IGHD5-4*01	IGHJ3*01	VH	Yes	Out-of-frame	No	-

V-(D)-J junction details based on top germline gene matches:

V region end	V-D junction*	D region	D-J junction*	J region start
GCTGC	TAAAATCTAACTGTGCAATACAGAGAACTACCTAGAT	AATACCTA	ACTCCAATCTTGAGGTATCTGTTGAAGAGGCTTTCAGCTATGAATTACCAAGAGGGATGCCTGTGTCTCCTACATCTTAAGGCATCTTTCTGACTACTATAGGTACGACGG	CCTGG

*: Overlapping nucleotides may exist at V-D-J junction (i.e, nucleotides that could be assigned
to either rearranging gene). Such nucleotides are indicated inside a parenthesis (i.e., (TACAT))
but are not included under the V, D or J gene itself.

Sub-region sequence details:

	Nucleotide sequence	Translation	Start	End
CDR3	AGATAATACCTAACTCCAATCTTGAGGTATCTGTTGAAGAGGCTTTCAGCTATGAATTACCAAGAGGGATGCCTGTGTCTCCTACATCTTAAGGCATCTTTCTGACTACTATAGGTACGACGGCCTGGTTTGCTTAC	R*YLTPILRYLLKRLSAMNYQEGCLCLLHLKASF*LL*VRRPGLLT	72	208

Alignment summary between query and top germline V gene hit:

	from	to	length	matches	mismatches	gaps	identity(%)
FR3-IMGT	8	38	31	29	2	0	93.5
Total			31	29	2	0	93.5

Alignments

```
                                                          <--------------------------FR3-IMGT--------------------------><------------------------
                                                            S  N  T  A  F  M  Q  L  S  C  *  N  L  T  V  Q  Y  R  E  L  P  R  *  Y  L  T  P  I  L  R
                       lcl|Query_1_reversed  8    CTCCAACACAGCCTTCATGCAGCTCAGCTGCTAAAATCTAACTGTGCAATACAGAGAACTACCTAGATAATACCTAACTCCAATCTTGAG  97
V  93.5% (29/31)   IGHV1-54*02           225  CTCCAACACAGCCTACATGCAGCTCAGCAGC-----------------------------------------------------------  255
                                                            S  N  T  A  Y  M  Q  L  S  S
V  90.3% (28/31)   IGHV1-55*01           225  CTCCAGCACAGCCTACATGCAGCTCAGCAGC-----------------------------------------------------------  255
V  90.3% (28/31)   IGHV1-62-1*01         225  CTCCAGCACAGCCTACATGCAGCTCAGCAGC-----------------------------------------------------------  255
D  100.0% (8/8)    IGHD5-2*01            2    --------------------------------------------------------------------AATACCTA--------------  9
D  100.0% (8/8)    IGHD5-3*01            2    --------------------------------------------------------------------AATACCTA--------------  9
D  100.0% (8/8)    IGHD5-4*01            2    --------------------------------------------------------------------AATACCTA--------------  9

                                                          --------------------------------------CDR3-IMGT------------------------------------------
                                                            Y  L  L  K  R  L  S  A  M  N  Y  Q  E  G  C  L  C  L  L  H  L  K  A  S  F  *  L  L  *  V
                       lcl|Query_1_reversed  98   GTATCTGTTGAAGAGGCTTTCAGCTATGAATTACCAAGAGGGATGCCTGTGTCTCCTACATCTTAAGGCATCTTTCTGACTACTATAGGT  187

                                                          -------------------->
                                                            R  R  P  G  L  L  T  G  A  K  G  L  W  S  L  S  L  Q
                       lcl|Query_1_reversed  188  ACGACGGCCTGGTTTGCTTACTGGGGCCAAGGGACTCTGGTCACTGTCTCTGCAG  242
J  100.0% (48/48)  IGHJ3*01              1    -------CCTGGTTTGCTTACTGGGGCCAAGGGACTCTGGTCACTGTCTCTGCAG  48
J  95.8% (46/48)   IGHJ3*02              1    -------CCTGGTTTGGTTAGTGGGGCCAAGGGACTCTGGTCACTGTCTCTGCAG  48
J  100.0% (14/14)  IGHJ2*01              12   ------------------TACTGGGGCCAAGG-----------------------  25
```

```
Lambda      K        H
    1.10    0.333    0.549

Gapped
Lambda      K        H
    1.08    0.280    0.540

Effective search space used: 64161651
```

```
Total queries = 1
Total identifiable CDR3 = 1
Total unique clonotypes = 1
```

Supplementary Figure 7: Analysis of the V(D)J junction sequences in *Pax5-het/Aid-het* and *Pax5-het/Aid-KO* B-ALL. Data showed that specific features regarding the junctions or choice of V, D, J segments were nor found. Sequence information for each band of interest is indicated in panel **b**) for band numbered as 1, panel **c**) for band numbered as 5, panel **d**) for band numbered as 4, and panel **e**) for band numbered as 6.

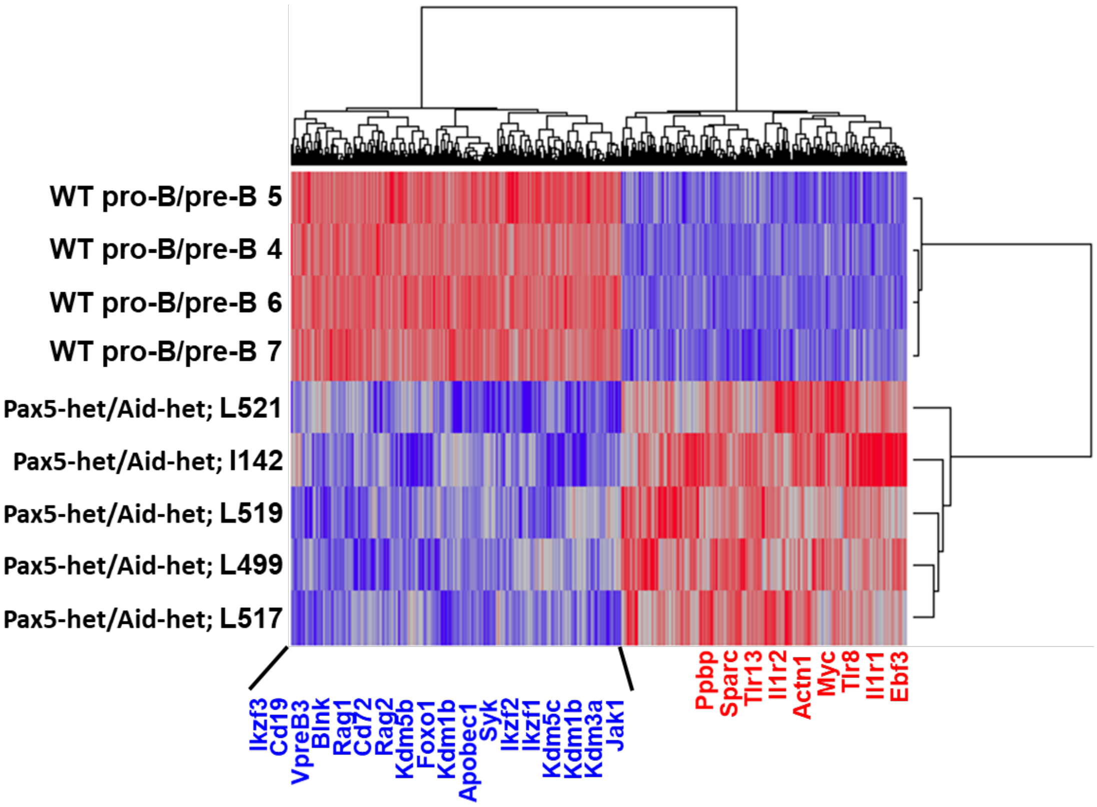

Supplementary Figure 8: Heat map of genes significantly induced or repressed within *Pax5-het/Aid-het* B-ALL. Genes significantly induced or repressed within tumor B cells of *Pax5-het/Aid-het* mice (L521, I142, L519, L499, and L517) in comparison with BM precursor pro/pre B-cells of wild-type mice (WT pro-B/pre-B 5, WT pro-B/pre-B 4, WT pro-B/pre-B 6, and WT pro-B/pre-B 7). as determined by significance analysis of microarrays using FDR 0.01% (7339 differentially expressed genes). Each row represents a separate gene, and each column denotes a separate mRNA sample. The level of expression of each gene in each sample is represented using a red–blue color scale (upregulated genes are displayed in red and downregulated genes in blue). Selected genes are highlighted based on their known relevance in infection-driven B-ALL.

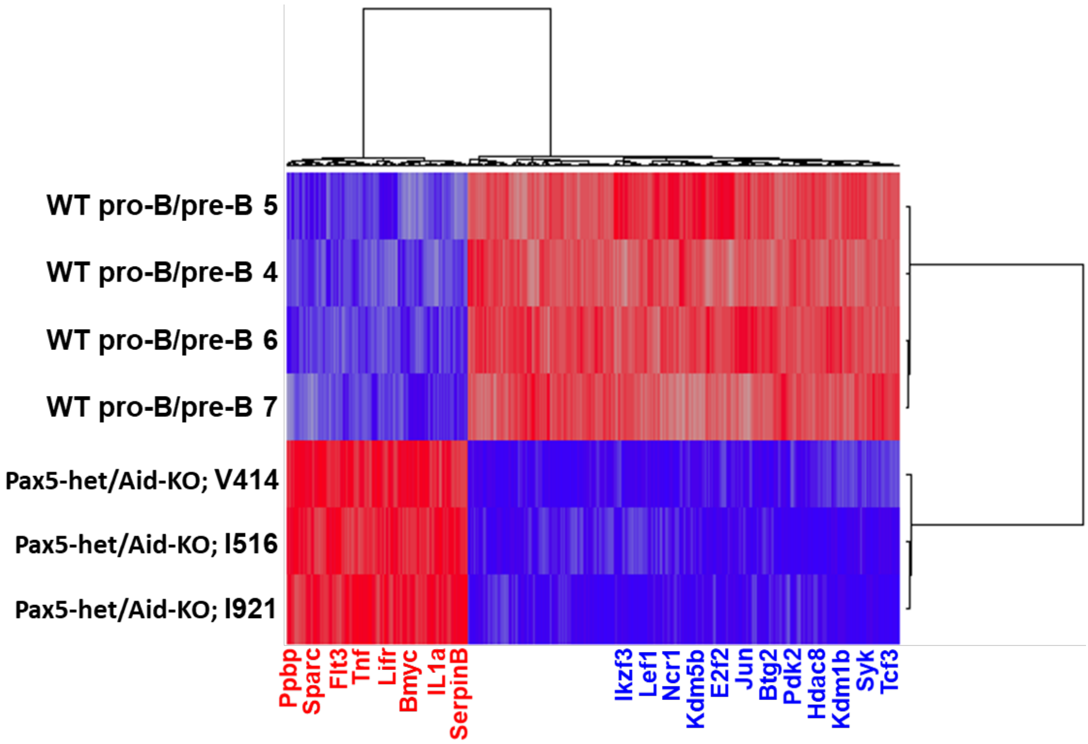

Supplementary Figure 9: Heat map of genes significantly induced or repressed within *Pax5-het/Aid-KO* B-ALL. Genes significantly induced or repressed within tumor B cells of *Pax5-het/Aid-KO* mice (V414, I516 and I921 in comparison with BM precursor pro/pre B-cells of wild-type mice (WT pro-B/pre-B 5, WT pro-B/pre-B 4, WT pro-B/pre-B 6, and WT pro-B/pre-B 7)., as determined by significance analysis of microarrays using FDR 0.01% (1053 differentially expressed genes). Each row represents a separate gene, and each column denotes a separate mRNA sample. The level of expression of each gene in each sample is represented using a red–blue color scale (upregulated genes are displayed in red and downregulated genes in blue). Selected genes are highlighted based on their known relevance in infection-driven B-ALL.

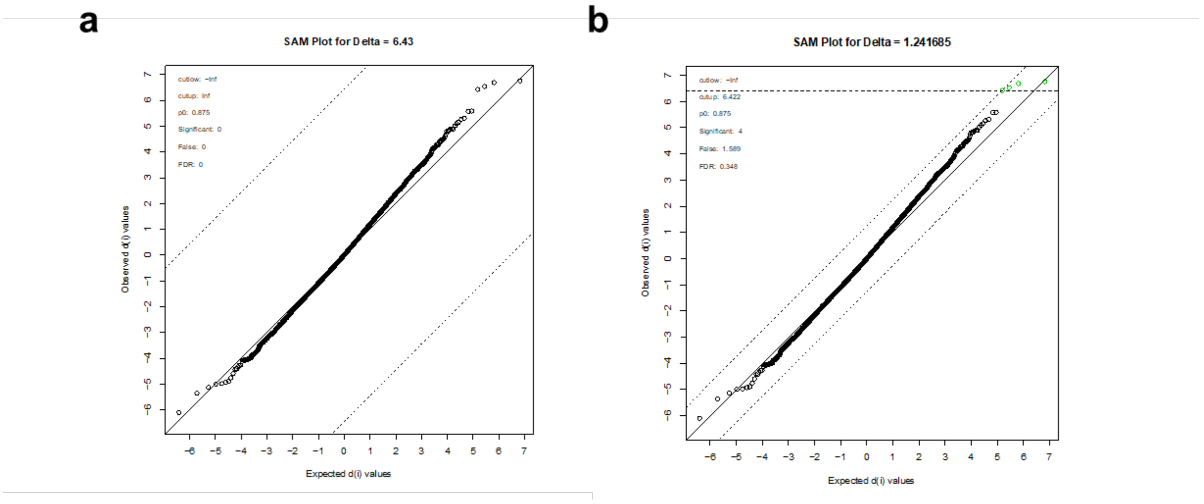

Supplementary Figure 10: Comparison of gene expression profiles between *Pax5-het/Aid-KO* B-ALL and *Pax5-het/Aid-het* B-ALL. a) SAM plot depicting the observed d-statistic versus the null distribution. The absence of a typical S-shape indicates that there is no significant difference between expression patterns in *Pax5-het/Aid-KO* B-ALL and *Pax5-het/Aid-het* B-ALL. **b)** The first cut-off that provide us with a set of overexpressed genes corresponds to a delta value of 1.241685 and a false discovery rate of 0.348 which is clearly beyond an acceptable statistical error.

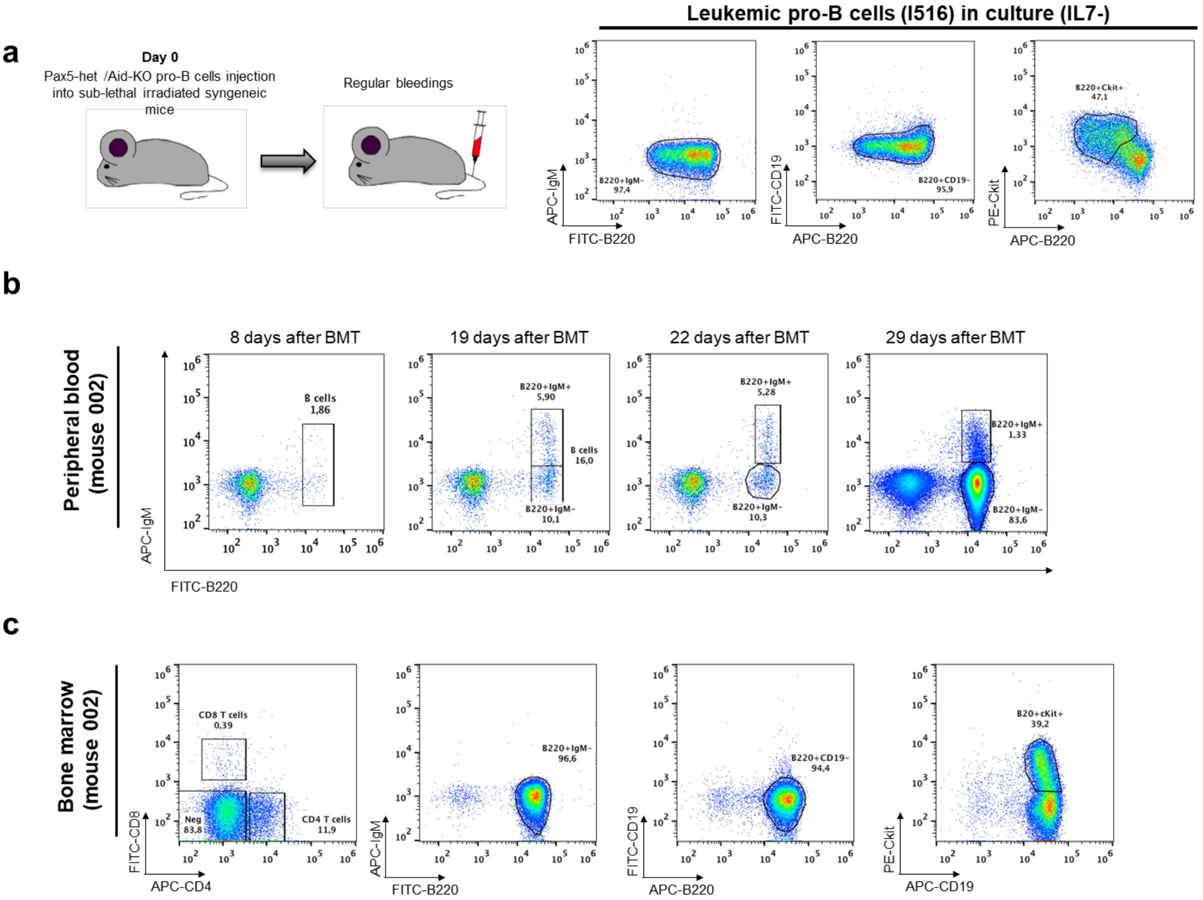

Supplementary Figure 11: *Pax5-het/Aid-KO* B-ALL is transplantable to secondary recipients. **a**, experimental setup. A total of 100,000 leukemic *Pax5-het/Aid-KO* pro-B cells mainatined in culture without IL-7 were injected into sublethally irradiated WT syngeneic mice. Regular bleedings were performed in order to monitor the development of the B-ALL. **b**, representative flow cytometric analysis of mice injected with leukemic *Pax5-het/Aid-KO* pro-B cells. Cytometric analyses at different time points show that leukemic B-ALL cells ($B220^{low}IgM^{-}$) were able to grow in secondary recipients. BMT, bone marrow transplant. **c**, representative flow cytometric analysis of mice injected with leukemic *Pax5-het/Aid-KO* pro-B cells shows the accumulation of B-ALL cells ($B220^{low}IgM^{-}$) in BM.

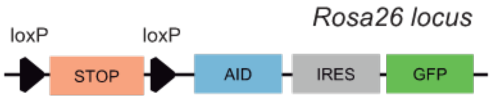

Supplementary Figure 12: Mouse model for conditional expression of Aid in B cells. Drawing of the construct used for conditional expression of Aid in B cells. An AID-IRES-GFP cassette preceded by a transcriptional STOP flanked by LoxP sites was introduced by homologous recombination within the endogenous Rosa26 locus (*R26*$^{+/Aid}$ *mice).*

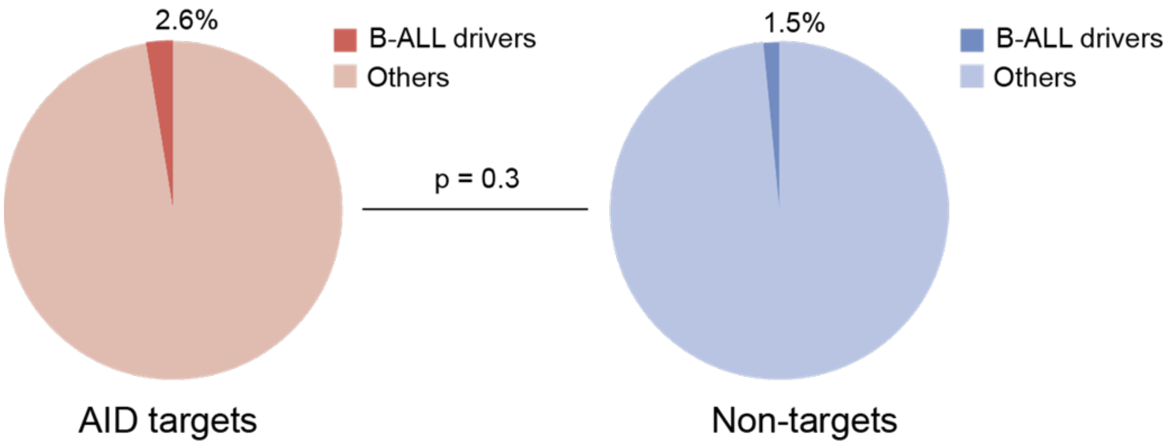

Supplementary Figure 13: AID targets are not enriched in B-ALL drivers. Piechart representation of the proportion of AID targets[1] (n=275, left) and non-target[1] (n=1104, right) that have been identified as B-ALL drivers[2] (n=59). Two-tailed Fisher test.

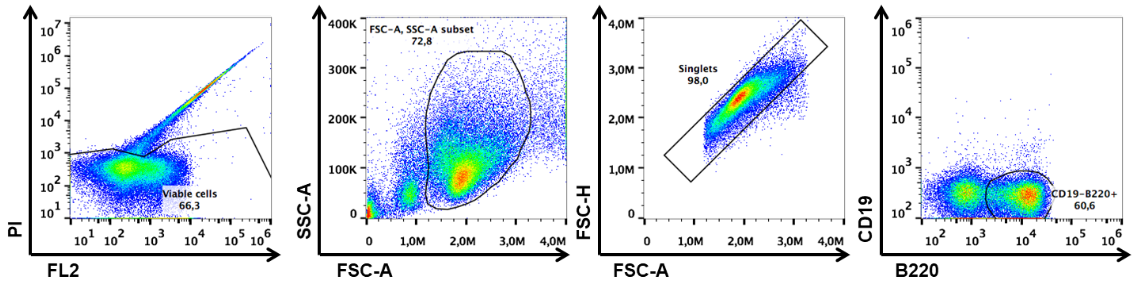

Supplementary Figure 14: Gating strategy used in FACS analysis. For each analysis, a total of at least 50,000 viable (PI-) cells were assessed. Singlets were selected prior gating strategy that is specific for each population analyzed. The same gating strategy has been used in all FACs analysis presented on Figure 1c, Supplementary Figure 2 and Supplementary Figure 11.

Supplementary Tables

Supplementary Table 1: SPF Facility Health Monitoring Report. The table shows the pathogens tested to monitor the health status of the animals housed in the SPF facility during the time the animals have been studied.

SPF FACILITY HEALTH MONITORING REPORT

Timeline	2015	2016	2017	2018	2019
**VIRUSES**					
MAD (Adenovirus type 1 and 2)	NEG	NEG	NEG	NEG	NEG
MCMV (Mouse cytomegalovirus)	NEG	NEG	NEG	NEG	NEG
LCMV (Lymph choriomeningitis)	NEG	NEG	NEG	NEG	NEG
Ectromelia	NEG	NEG	NEG	NEG	NEG
EDIM	NEG	NEG	NEG	NEG	NEG
Hantaan virus	NEG	NEG	NEG	NEG	NEG
MHV (Mouse hepatitis virus)	NEG	NEG	NEG	NEG	NEG
MVM (Minute virus)	NEG	NEG	NEG	NEG	NEG
MPV (Parvovirus type 1 and 2)	NEG	NEG	NEG	NEG	NEG
PVM (Pneumonia virus)	NEG	NEG	NEG	NEG	NEG
Reovirus type 3	NEG	NEG	NEG	NEG	NEG
Sendai	NEG	NEG	NEG	NEG	NEG
TMEV (Theiler´s murine encephalomyel)	NEG	NEG	NEG	NEG	NEG
K virus	NEG	NEG	NEG	NEG	NEG
Polyoma virus	NEG	NEG	NEG	NEG	NEG
**BACTERIA**					
Bordetella bronchiseptica	NEG	NEG	NEG	NEG	NEG
Car-bacillus	NEG	NEG	NEG	NEG	NEG
Citrobacter rodentium	NEG	NEG	NEG	NEG	NEG
Clostridium piliforme (Tyzzer´ disease)	NEG	NEG	NEG	NEG	NEG
Corynebacterium kutscheri	NEG	NEG	NEG	NEG	NEG
Mycoplasma pulmonis	NEG	NEG	NEG	NEG	NEG
Pasteurella pneumotropica	NEG	NEG	NEG	NEG	NEG
Pasteurella multocida	NEG	NEG	NEG	NEG	NEG
Salmonella spp	NEG	NEG	NEG	NEG	NEG
Streptobacillus moniliformis	NEG	NEG	NEG	NEG	NEG
Streptococci beta hemolíticos (A,G)	NEG	NEG	NEG	NEG	NEG
Streptococcus pneumoniae	NEG	NEG	NEG	NEG	NEG
Helicobacter spp	NEG	NEG	NEG	NEG	NEG
**PARASITES**					
Ectoparasites	NEG	NEG	NEG	NEG	NEG
Helminth (A.tetraptrera, Syphacia spp)	NEG	NEG	NEG	NEG	NEG
Cestods (Hymenolepis spp)	NEG	NEG	NEG	NEG	NEG
Eimeria spp	NEG	NEG	NEG	NEG	NEG
Giardia spp	NEG	NEG	NEG	NEG	NEG
Trichomonas muris	NEG	NEG	NEG	NEG	NEG
Spironucleus spp	NEG	NEG	NEG	NEG	NEG
Encephalitozoon cuniculi	NEG	NEG	NEG	NEG	NEG
**NECROPSY**	No gross lesion.	No gross lesion.	No gross lesion.	No gross lesion.	No gross lesion.

NEG: Negative
POS: Positive

SPF: specific pathogen free

Supplementary Table 2: Conventional Facility Health Monitoring Report. The table shows the pathogens tested to monitor the health status of the animals housed in the conventional facility during the time the animals have been studied. Indicated are pathogens to which the mice were exposed when transferred to the conventional animal facility.

CONVENTIONAL FACILITY HEALTH MONITORING REPORT					
**Timeline**	**2015**	**2016**	**2017**	**2018**	**2019**
**VIRUSES**					
MAD (Adenovirus type 1 and 2)	NEG	NEG	NEG	NEG	NEG
MCMV (Mouse cytomegalovirus)	NEG	NEG	NEG	NEG	NEG
LCMV (Lymph choriomeningitis)	NEG	NEG	NEG	NEG	NEG
Ectromelia	NEG	NEG	NEG	NEG	NEG
EDIM	NEG	NEG	NEG	NEG	NEG
Hantaan virus	NEG	NEG	NEG	NEG	NEG
MHV (Mouse hepatitis virus)	POS	POS	POS	POS	POS
MVM (Minute virus)	NEG	NEG	NEG	NEG	NEG
MPV (Parvovirus type 1 and 2)	NEG	NEG	NEG	NEG	NEG
PVM (Pneumonia virus)	NEG	NEG	NEG	NEG	NEG
Reovirus type 3	NEG	NEG	NEG	NEG	NEG
Sendai	NEG	NEG	NEG	NEG	NEG
TMEV (Theiler´s murine encephalomyel)	NEG	NEG	NEG	NEG	NEG
K virus	NEG	NEG	NEG	NEG	NEG
Polyoma virus	NEG	NEG	NEG	NEG	NEG
**BACTERIA**					
Bordetella bronchiseptica	NEG	NEG	NEG	NEG	NEG
Car-bacillus	NEG	NEG	NEG	NEG	NEG
Citrobacter rodentium	NEG	NEG	NEG	NEG	NEG
Clostridium piliforme (Tyzzer´ disease)	NEG	NEG	NEG	NEG	NEG
Corynebacterium kutscheri	NEG	NEG	NEG	NEG	NEG
Mycoplasma pulmonis	NEG	NEG	NEG	NEG	NEG
Pasteurella pneumotropica	NEG	NEG	NEG	NEG	NEG
Pasteurella multocida	NEG	NEG	NEG	NEG	NEG
Salmonella spp	NEG	NEG	NEG	NEG	NEG
Streptobacillus moniliformis	NEG	NEG	NEG	NEG	NEG
Streptococci beta hemolíticos (A,G)	NEG	NEG	NEG	NEG	NEG
Streptococcus pneumoniae	NEG	NEG	NEG	NEG	NEG
Helicobacter spp	POS	POS	POS	POS	POS
**PARASITES**					
Ectoparasites	NEG	NEG	NEG	NEG	NEG
Helminth (A.tetraptrera, Syphacia spp)	POS	POS	POS	POS	POS
Cestods (Hymenolepis spp)	NEG	NEG	NEG	NEG	NEG
Eimeria spp	NEG	NEG	NEG	NEG	NEG
Giardia spp	NEG	NEG	NEG	NEG	NEG
Trichomonas muris	POS	POS	POS	POS	POS
Spironucleus spp	NEG	NEG	NEG	NEG	NEG
Encephalitozoon cuniculi	NEG	NEG	NEG	NEG	NEG
**NECROPSY**	No gross lesion.	No gross lesion.	No gross lesi	No gross lesion.	No gross lesion.

**NEG: Negative**
**POS: Positive**

Supplementary Table 3. List of cancer-related genes mutated in *Pax5-het/Aid-het* and *Pax5-het/Aid-KO* B-ALL, their mutational context highlighting if they are located in AID hotspots or not.

Mouse genotype	Mouse ID	Gene	Position	6 nucleotides before	ref	mut	6 nucleotides after	Prot. Consequence	AID hotspot
Pax5-het/Aid-het	L503	Pax5	4:44692006	ACCCCC	G	C	GCTTGA	P80R	-
	L503	Jak1	4:101157867	GGCTTC	A	G	GGGACT	L909P	-
	L503	Jak3	8:71684008	TGGCTC	G	A	TGAGGG	R653H	-
	L519	Pax5	4:44692006	ACCCCC	G	C	GCTTGA	P80R	-
	L519	Trp53	11:69588512	TGAGAC	G	A	CTGCCC	R169H	-
	L499	Ptpn11	5:121143606	ATGAAG	G	A	TTCCTG	T468I	RGYW
	L499	Ezh2	6:47532941	ATTTGT	C	T	ATACAC	D659N	-
	L521	Tsc2	17:24599594	TGGAGG	C	A	CGCAGC	A1424S	-
Pax5-het/Aid-KO	I921	Fancm	12:65113969	AAAACA	A	T	CGAAGT	N1407I	WA
	I921	Pax5	4:44691991	TTGGAT	C	G	CTCCAA	G85A	-
	I516	Ptpn11	5:121143097	ATCCCC	G	A	ACCTCT	S502L	-
	V414	Ptpn11	5:121167955	CCAGTT	C	T	AGCCAA	E76K	-
	V414	Trp53	11:69589214	ACCGCC	G	A	ACCTAT	R243Q	-
	V517	Nras	3:103060272	TGGACA	A	C	GAGGAG	Q61H	WA

Supplementary Table 4: List of human B-ALL drivers[1] and the mutational status of their mouse orthologs in *Pax5-het/Aid-het* and *Pax5-het/Aid-KO* B-ALL. First column indicates whether these driver genes are AID off-targets in mouse[2].

B-ALL drivers	AID-off target	Mutated in *Pax5-het/Aid-het*	Mutated in *Pax5-het/Aid-KO*	Mouse_ortolog
ABL1	-	-	-	Abl1
ADD3	-	-	-	Add3
ARID2	-	-	-	Arid2
ASXL1	-	-	-	Asxl1
ATF7IP	-	-	-	Atf7ip
ATRX	-	-	-	Atrx
BCORL1	-	-	-	Bcorl1
BTG1	Yes	-	-	Btg1
CBL	-	-	-	Cbl
CCND3	-	-	-	Ccnd3
CD200	-	-	-	Cd200
CDKN2A	-	-	-	Cdkn2a
CHD4	-	-	-	Chd4
CREBBP	-	-	-	Crebbp
CRLF2	-	-	-	Crlf2
CTCF	-	-	-	Ctcf
EBF1	Yes	-	-	Ebf1
ELF1	-	-	-	Elf1
EP300	-	-	-	Ep300
ERG	-	-	-	Erg
ETV6	-	-	-	Etv6
FLT3	-	-	-	Flt3
IKZF1	Yes	-	-	Ikzf1
IKZF3	-	-	-	Ikzf3
IL7R	-	-	-	Il7r
INO80	-	-	-	Ino80
JAK1	-	Yes	-	Jak1
JAK2	-	-	-	Jak2
KMT2A	-	-	-	Kmt2a
KMT2D	-	-	-	Kmt2d
KRAS	-	-	-	Kras
LEMD3	-	-	-	Lemd3
MEF2D	-	-	-	Mef2d
MGA	-	-	-	Mga
MYC	Yes	-	-	Myc
NF1	-	-	-	Nf1
NRAS	-	-	Yes	Nras
PAX5	Yes	Yes	Yes	Pax5
PHF6	-	-	-	Phf6
PTPN11	-	Yes	Yes	Ptpn11
RAG1	-	-	-	Rag1
RAG2	-	-	-	Rag2
RB1	-	-	-	Rb1
RUNX1	-	-	-	Runx1
SETD2	-	-	-	Setd2
SH2B3	-	-	-	Sh2b3
TBL1XR1	-	-	-	Tbl1xr1
TCF3	Yes	-	-	Tcf3
TOX	-	-	-	Tox
TP53	-	Yes	Yes	Trp53
UBA2	-	-	-	Uba2
USP9X	-	-	-	Usp9x
WAC	-	-	-	Wac
WHSC1	-	-	-	Nsd2
XBP1	Yes	-	-	Xbp1
ZFP36L2	-	-	-	Zfp36l2
ZMIZ1	-	-	-	Zmiz1
ZFP217	-	-	-	Zfp217
ZFP384	-	-	-	Zfp384

Supplementary Table 5: V(D)J junction sequences information in *Pax5-het/Aid-het* and *Pax5-het/Aid-KO* B-ALL

Sequence ID	PRIMER	PCR Band number	Band size	Mouse ID
CVD001	VH7183 (Forward)	1	359bp	2-I516 (*Pax5-het/Aid-KO*)
CVD002	JH3 (Reverse)			
CVD005	DJ (Forward)	3	1,4Kb	2-I516 (*Pax5-het/Aid-KO*)
CVD006	JH3 (Reverse)			
CVD007	VH7183 (Forward)	4	1,1Kb	4-L503 (*Pax5-het/Aid-het*)
CVD008	JH3 (Reverse)			
CVD009	VHQ52 (Forward)	5	793bp	6-L517 (*Pax5-het/Aid-het*)
CVD010	JH3 (Reverse)			
CVD011	VHJ558 (Forward)	6	465bp	5-I142 (*Pax5-het/Aid-het*)
CVD012	JH3 (Reverse)			

Supplementary References

1. Alvarez-Prado AF*, et al.* A broad atlas of somatic hypermutation allows prediction of activation-induced deaminase targets. *J Exp Med* **215**, 761-771 (2018).

2. Ma X*, et al.* Pan-cancer genome and transcriptome analyses of 1,699 paediatric leukaemias and solid tumours. *Nature* **555**, 371-376 (2018).
