# Supplementary material for: Infectious stimuli promote malignant B-cell acute lymphoblastic leukemia in the absence of AID
Source: Nat Commun. 2019 Dec 5;10:5563. doi: 10.1038/s41467-019-13570-y (PMC6895129; doi:10.1038/s41467-019-13570-y)
Supplement: Supplementary file 3 — Description of Additional Supplementary Files [file 41467_2019_13570_MOESM3_ESM.pdf]

Description of Additional Supplementary Files

File Name: Supplementary Data 1
Description: Differentially expressed genes in Pax5-het/Aid-het BALL compared to BM pro/pre B cells from wild-type mice.

File Name: Supplementary Data 2
Description: Differentially expressed genes in Pax5-het/Aid-KO BALL compared to BM pro/pre B cells from wild-type mice.
