# Supplementary material for: Infectious stimuli promote malignant B-cell acute lymphoblastic leukemia in the absence of AID
Source: Nat Commun. 2019 Dec 5;10:5563. doi: 10.1038/s41467-019-13570-y (PMC6895129; doi:10.1038/s41467-019-13570-y)
Supplement: Supplementary file 6 — Reporting Summary [file 41467_2019_13570_MOESM6_ESM.pdf]

natureresearch

Corresponding author(s): DBPR (NCOMMS-19-07109D)

Last updated by author(s): Nov 6, 2019

Reporting Summary

Nature Research wishes to improve the reproducibility of the work that we publish. This form provides structure for consistency and transparency in reporting. For further information on Nature Research policies, see Authors & Referees and the Editorial Policy Checklist.

Statistics

For all statistical analyses, confirm that the following items are present in the figure legend, table legend, main text, or Methods section.

n/a	Confirmed	
☐	☒	The exact sample size (*n*) for each experimental group/condition, given as a discrete number and unit of measurement
☐	☒	A statement on whether measurements were taken from distinct samples or whether the same sample was measured repeatedly
☐	☒	The statistical test(s) used AND whether they are one- or two-sided<br>*Only common tests should be described solely by name; describe more complex techniques in the Methods section.*
☒	☐	A description of all covariates tested
☒	☐	A description of any assumptions or corrections, such as tests of normality and adjustment for multiple comparisons
☐	☒	A full description of the statistical parameters including central tendency (e.g. means) or other basic estimates (e.g. regression coefficient) AND variation (e.g. standard deviation) or associated estimates of uncertainty (e.g. confidence intervals)
☐	☒	For null hypothesis testing, the test statistic (e.g. *F*, *t*, *r*) with confidence intervals, effect sizes, degrees of freedom and *P* value noted<br>*Give P values as exact values whenever suitable.*
☒	☐	For Bayesian analysis, information on the choice of priors and Markov chain Monte Carlo settings
☒	☐	For hierarchical and complex designs, identification of the appropriate level for tests and full reporting of outcomes
☒	☐	Estimates of effect sizes (e.g. Cohen's *d*, Pearson's *r*), indicating how they were calculated

Our web collection on statistics for biologists contains articles on many of the points above.

Software and code

Policy information about availability of computer code

Data collection	BD AccuriTM C6 Flow Cytometer for FACs analysis. TruSeq SBS Kit v3 on the HiSeq 2500 (Illumina) for sequencing
Data analysis	Software and Algorithms: FlowJo (version 10.1.r7), R (R Core Team, https://www.R-project.org/), GSEA (Broad Institute, http://software.broadinstitute.org/gsea/index.jsp), GraphPad Prism (version 6.0-GraphPad Software Inc), BcltoFastq 1.8.4 (Illumina), BWA version 0.7.4., GATK 2.4.9. Mouse dbSNP138, Ensembl database (v70), MuTect and VarScan, Ensembl's BioMart.

For manuscripts utilizing custom algorithms or software that are central to the research but not yet described in published literature, software must be made available to editors/reviewers. We strongly encourage code deposition in a community repository (e.g. GitHub). See the Nature Research guidelines for submitting code & software for further information.

Data

Policy information about availability of data

All manuscripts must include a data availability statement. This statement should provide the following information, where applicable:

- Accession codes, unique identifiers, or web links for publicly available datasets
- A list of figures that have associated raw data
- A description of any restrictions on data availability

Authors can confirm that all relevant data are included in the paper and/or its supplementary information files. Gene Expression Data are accessible through GEO Series accession number GSE122105. The source data underlying Extended Data Figure 6 is provided as a Source Data file.

Field-specific reporting

Please select the one below that is the best fit for your research. If you are not sure, read the appropriate sections before making your selection.

☒ Life sciences ☐ Behavioural & social sciences ☐ Ecological, evolutionary & environmental sciences

For a reference copy of the document with all sections, see nature.com/documents/nr-reporting-summary-flat.pdf

Life sciences study design

All studies must disclose on these points even when the disclosure is negative.

Sample size	The animal numbers were calculated based on the following assumptions and taking into account the special working hypothesis. 30 final experimental mice per group is the minimal number allowing the use of parametrical statistical tests. According to power analysis calculations, assuming development of ALL as the experimental endpoint and an expected proportion of 0.05 leukaemia-free-exposed mice
Data exclusions	Not applicable. The inclusion criteria was based on the genotype of the mouse: transgenic versus control littermate.
Replication	All experiments have been conducted at least three times to ensure reproducibility.
Randomization	Not applicable. The inclusion criteria was based on the genotype of the mouse: transgenic versus control littermate.
Blinding	The investigator was not blinded as the inclusion criteria was based on the genotype of the mouse: transgenic versus control littermate.

Reporting for specific materials, systems and methods

We require information from authors about some types of materials, experimental systems and methods used in many studies. Here, indicate whether each material, system or method listed is relevant to your study. If you are not sure if a list item applies to your research, read the appropriate section before selecting a response.

Materials & experimental systems

n/a	Involved in the study	
☐	☒	Antibodies
☒	☐	Eukaryotic cell lines
☒	☐	Palaeontology
☐	☒	Animals and other organisms
☒	☐	Human research participants
☒	☐	Clinical data

Methods

n/a	Involved in the study	
☒	☐	ChIP-seq
☐	☒	Flow cytometry
☒	☐	MRI-based neuroimaging

Antibodies

Antibodies used	The following antibodies were used for flow cytometry: anti-B220 (RA3-6B2), CD3Ɛ (145-2C11), CD4 (RM4-5, 1:250), CD8a (53-6.7, 1:250), CD11b/Mac1 (M1/70, 1:200), CD19 (1D3), CD117/c-Kit (2B8, 1:200), CD127/IL-7Rα (A7R34, 1:50), Ly-6G/Gr1 (RB6-8C5), IgM (R6-60.2), Sca1/Ly-6A/E (E13-161.7, 1:50), CD25 (PC61), CD48 (HM48-1) and CD150 (TC15-12F12.2) antibodies. Unspecific antibody binding was suppressed by preincubation with CD16/CD32 (2.4G2) Fc-block solution (PharMingen). All antibodies were purchased from BD Biosciences and used at a 1:100 dilutions unless otherwise indicated.
Validation	All the antibodies has been previously used in relevant publications as for example: EMBO J. 2018 Jul 13;37(14); Cancer Res. 2018 May 15;78(10):2669-2679; Cancer Res. 2017 Aug 15;77(16):4365-4377 and Cancer Discov. 2015 Dec;5(12):1328-43.

Animals and other organisms

Policy information about studies involving animals; ARRIVE guidelines recommended for reporting animal research

Laboratory animals	Animal models used were mice. Both male and female Aid-het, Aid-KO, Pax5-het/Aid-het, Pax5-het/Aid-KO, Rosa26AID knock-in mice, Mb-1cre, P53-/- and Ink4/Arf-/- mice of a mixed C57BL/6 x CBA background were included in the study.
Wild animals	Not applicable.
Field-collected samples	We confirm that all aspects of animal studies have been adequately reported. The housing conditions are detail in the manuscript as supplementary information.
Ethics oversight	All animal work has been conducted according to relevant national and international guidelines and it has been approved by the

Ethics oversight	Bioethics Committee of University of Salamanca and by the Bioethics Subcommittee of Consejo Superior de Investigaciones Cientificas (CSIC).

Note that full information on the approval of the study protocol must also be provided in the manuscript.

Flow Cytometry

Plots

Confirm that:

☒ The axis labels state the marker and fluorochrome used (e.g. CD4-FITC).

☒ The axis scales are clearly visible. Include numbers along axes only for bottom left plot of group (a 'group' is an analysis of identical markers).

☒ All plots are contour plots with outliers or pseudocolor plots.

☒ A numerical value for number of cells or percentage (with statistics) is provided.

Methodology

Sample preparation	Nucleated cells were obtained from total mouse bone marrow (flushing from the long bones), peripheral blood, thymus, or spleen. Contaminating red blood cells were lysed with RCLB lysis buffer and the remaining cells were washed in PBS with 1% FCS. After staining, all cells were washed once in PBS and were resuspended in PBS with 1% FCS containing 10 �g/mL propidium iodide (PI) to allow dead cells to be excluded from both analyses and sorting procedures.
Instrument	The samples and the data were acquired in an AccuriC6 Flow Cytometer.
Software	The samples were analyzed using Flowjo software (version 10.1.r7)
Cell population abundance	The purity of the samples post-sorting was determined by acquiring 50.000 events from the sorted population. For all cases the sorted population had around 95% of purity.
Gating strategy	For each analysis, a total of at least 50,000 viable (PI-) cells were assessed. Singlets were selected prior gating strategy that is specific for each population anlalyzed and shown in the figures of the manuscript.

☒ Tick this box to confirm that a figure exemplifying the gating strategy is provided in the Supplementary Information.
